# *Āsana* for Neck, Shoulders, and Wrists to Prevent Musculoskeletal Disorders among Dental Professionals: In-Office Yóga Protocol

**DOI:** 10.3390/jfmk8010026

**Published:** 2023-02-20

**Authors:** Maria Giovanna Gandolfi, Fausto Zamparini, Andrea Spinelli, Carlo Prati

**Affiliations:** 1 Ergonomics, Posturology and Yóga Therapy Program, Degree in Dentistry and Degree Course in Dental Hygiene, School of Medicine, University of Bologna, 40125 Bologna, Italy; 2 Yóga Therapy Program, Specialization in Sports Medicine, School of Medicine, University of Bologna, 40125 Bologna, Italy; 3 Department of Biomedical and Neuromotor Sciences, Dental School, University of Bologna, 40125 Bologna, Italy

**Keywords:** musculoskeletal disorders, neck pain, chronic headache, shoulder pain, compressive syndromes, carpal tunnel syndrome, outlet syndromes, impingement syndrome, spinal disc pathologies, Yoga therapy, *Parināma Yoga*, *āsana*, dental professionals, dentists, dental hygienists, dental ergonomics, occupational risk factors, preventive exercises

## Abstract

Extensive literature reports an increase in physical disorders (pain, pathologies, dysfunctions) and mental malaise/uneasiness (stress, burnout) affecting dental professionals in relation to fast and pressing rhythms of work, long working hours, increasingly demanding patients, ever-evolving technologies, etc. This project has been conceived to bring the science of yoga around the world to dental professionals as a preventive (occupational) medicine and to provide knowledge and means for self-care. Yoga is a concentrative self-discipline of the mind, senses, and physical body, that requires regular daily exercise (or meditation), attention, intention, and disciplined action. **M&M**: The study aimed to design a Yoga protocol specifically devised for dental professionals (dentists, dental hygienists, and dental assistants) including positions (*āsana*) to be practiced/used in the dental office. The protocol is targeted for the upper body, namely neck, upper back, chest, shoulder girdle, and wrists, being areas greatly affected by work-related musculoskeletal disorders. This paper represents a yoga-based guideline for the self-cure of musculoskeletal disorders among dental professionals. **Results**: The protocol includes both sitting (*Upavistha* position) and standing (*Utthana* or *Sama* position) *āsana*, with twisting (*Parivrtta*), side bending (*Parsva*), flexion and forward bending (*Pashima*), and extension and arching (*Purva*) *āsana* to mobilize and decompress, and to provide nourishment and oxygen to the musculo-articular system. The paper delivers different concepts and theories developed and deepened by the authors and introduces and spreads yoga as a medical science among dental professionals for the prevention and treatment of work-related musculoskeletal disorders. We articulate notions ranging from stretching out using the *vinyāsa* method (breath-driven movement) and inward-focused attention to contemplative/concentrative science, interoceptive attention, self-awareness, the mind–body connection, and receptive attitude. The theory of “muscles are bone ties” is coined and delivered with regard to tensegrity musculoskeletal fascial structures connecting, pulling together, and nearing the bone segments where they are anchored. The paper describes over 60 *āsana* envisaged to be performed on dental stools or using the walls of a dental office or a dental unit chair. A detailed guideline on the work-related disorders that can find relief with the protocol is provided, including the description of breath control for the practice of *āsana* in *vinyāsa*. The foundations of the technique reside in the *Iyengar Yoga* method and *Parināma Yoga* method. **Conclusions**: This paper represents a guideline for self-cure in the prevention or treatment of musculoskeletal disorders affecting dental professionals. Yoga is a powerful concentrative self-discipline able to provide physical and mental well-being, representing great help and support in daily life and business for dental professionals. *Yógāsana* restores retracted and stiff muscles, giving relief to the strained and tired limbs of dental professionals. Yoga is not intended for flexible or physically performing persons but for people who decide to take care of themselves. The practice of specific *āsana* represents a powerful tool for the prevention or treatment of MSDs related to poor posture, forward head, chronic neck tension (and related headache), depressed chest, compressive disorders on wrists and shoulders as carpal tunnel, impingement syndromes, outlet syndrome, subacromial pain syndrome and spinal disc pathologies. Yoga, as an integrative science in medicine and public health, represents a powerful tool for the prevention and treatment of occupational musculoskeletal disorders and an extraordinary path for the self-care of dental professionals, sitting job workers, and healthcare providers suffering from occupational biomechanical stresses and awkward postures.

## 1. Introduction

A substantial literature reports growing physical and psychological/mental disorders affecting dental professionals [1,2,3]. Long working hours, a fast pace of work, pressing and fastened working, increasingly demanding patients, increasingly difficult accounting management, complicated and time-consuming interactions with external laboratories, relationships with colleagues, etc., subject dental professionals to increasing malaise/uneasiness, mental (stress, burnout) and physical (pain) pathologies, and dysfunctions.

Diverse literature published in high-ranking medical journals reports that the neck and shoulder girdle are among the areas most affected by musculoskeletal disorders in dental professionals [2,3,4,5,6,7]. Muscle overuse, repetitive movements, emotional stress, poor posture, lack of muscular and articular mobility, nerve pinching, or compression can cause muscular pain, paresthesia, tingling, muscle weakness/soreness/spasms/cramps, and muscle injury. Trigger points form when segments of muscle fibers remain stuck in a contracted state [3]. The most affected muscles are in the upper back, shoulders, and neck, and involve the sternocleidomastoids, trapezius, levator scapulae, infraspinatus, and rhomboids.

Several syndromes often affecting dental professionals are carpal tunnel syndrome, shoulder disorders, tension headache, compressive syndromes and neuropathies, thoracic and shoulder outlet syndromes, and impingement syndromes [3].

Considering that this paper is intended for people who superficially know or ignore yoga, it is important to briefly report historical sources on its essence and significance.

Yoga is a system of physical, mental, and spiritual techniques or practices developed to control (yoke) and still the mind, providing well-being. Yoga is the science of body–mind contact, the science of mind embodiment, an endless path of psycho-physical self-research and development. *Yogāsana* practice connects the movement of the body and the fluctuations of the mind to the rhythm of our breath. Yoga educates us to our inward attention, to feel our body, to recognize our habitual thought patterns, to cultivate awareness from moment to moment. 

Historical (premedieval) definitions of yoga refer to knowledge, calming, and blooming of the self.

Yoga is “mind in the self” (Vaiśeṣika Sūtra, 4th century BCE), “when … the mind remain still” (Kata Upanishad, last centuries BCE); “yoga is skill in action” and “yoga is the journey of the self, through the self, to the self” (Bhagavad Gita, second century BCE); “yoga is the calming down the fluctuations of mind” and “yoga is the stilling of the changing states of the mind” (Yoga Sutras of Patanjali, first centuries CE); “yoga is the means of perceiving reality” (Brahma Sutra Bhasya of Adi Shankararya, 8th century CE).

Yoga as precious source of calm, well-being, lucidity, and health, has been object of study in different medical disciplines. Yoga as a medical science has received increasing attention over the past decades.

The high number of top-ranked scientific publications in different medical areas has placed yoga among the medical approaches, owing to its demonstrated therapeutic effects and benefits for both physical and mental/cognitive health.

Many clinical studies on yoga as medical science have been published in the top-ranking journals of various medical fields, such as cardiology for cardiovascular diseases [8,9,10], coronary heart disease [11], hypertension [12], atrial fibrillation and arrhythmia [13] and vasovagal syncope [14]; internal medicine for metabolic syndrome [10,15], irritable bowel [16], inflammatory markers [17,18], and the immune system [19]; pneumology [20,21]; orthopedics/physiatry for chronic pain [22,23,24,25] and disc herniation [26,27,28]; neurology/psychology for stress, anxiety, and burnout [29,30]; and occupational and preventive medicine and public health [30,31].

This paper aims to design a Yoga protocol specifically tailored for dental professionals (dentists, dental hygienists, and dental assistants) to be practiced/used on dental stools or using the walls of a dental office or a dental unit chair. The protocol is targeted to the neck, shoulder girdle and wrists among the areas greatly affected by musculoskeletal disorders. The proposed *āsana* include movements in all directions of space to mobilize and decompress the musculo-articular system.

## 2. Materials and Methods

This study aims to design a protocol focused on the mobilization, loosening up, and decompression of the wrist/hand and neck and shoulder articulations.

### 2.1. Targeted Body Areas

The protocol intends to seek/search among yoga postures and identify *āsana*-derived positions and movements finalized to be practiced using a dental stool and the structures present in the dental office (dental chair, clinic/office walls, and furniture).

The targeted body areas involved in the conception of the yoga positions are:

- Wrist joints (radioulnar, radiocarpal, and midcarpal joints);
- Hand/finger joints (metacarpophalangeal and interphalangeal joints);
- Cranio-cervical junction (atlanto-occipital and atlas-axis joints);
- Shoulder girdle (gleno-humeral joint, sterno-clavicular joint, acromio-clavicular joint, scapula-thoracic joint);
- Intervertebral articulations.

*Āsana* and the Yoga protocol were conceived of by an experienced registered yoga teacher (M.G.G.) with over 20 years of daily self-practice, over than 1200 h of experience in teacher training courses and over 10 years teaching in institutional university courses. The protocol was contrived with a rationale of movements in all directions of space, with gradually increasing intensity. The origin to insertion of the muscles involved in the described movements have been enlisted throughout the text in parentheses as (O to I).

### 2.2. Execution Instructions/Recommendations

The authors recommend—as a foundation for postures in Yoga therapy—that movements be aware, slow, and progressive, and always performed by setting the intention to activate body thrust in three spatial directions (three-dimensional push) with the intention of steady active pushing in three divergent directions. 

We want to provide the concept that active pushing is basically different from active stretching.

The Yoga protocol includes both sitting and standing positions, with twisting, flexions, extensions, forward bends, and arching for musculo-articular decompression and mobilization. The protocol entails that the practice of the *āsana* is in a state of concentration and in *vinyāsa* (movements flowing with breath). Enter into each *āsana* slowly and gently, possibly with closed or half-closed eyes and the intention to stretch away, to extend, and to expand outward with a three-dimensional deep extension. Create the expansion by pushing (not pulling) your limbs away. For each inhalation, search for an increase in your inner space; for each exhalation, search for an extension of the stretch. Remain open and receptive, keep a serendipity mindset, and welcome every feeling without judgement or opposition or resistance, as if you had to melt into the position. When the tension is strong or painful, keep your focus on slow even breathing and, if necessary, reduce the stretch/extension slightly.

We would like to permeate the mind with the concept that yoga gives well-being and serendipity, i.e., unexpected sensations and messages from our body. 

A main teaching from intense *āsana* is to train our mind to be comfortable in discomfort, to be calm and still in an uneasy, difficult, or painful state/situation.

### 2.3. Āsana (Yoga Postures)

Over 60 yoga-derived postures have been proposed to mobilize and decompress the musculoskeletal structures of dental professionals during their daily routine in the dental office.

The authors propose and warmly suggest short breaks of 15–30 min (at least at mid-morning, at lunch time, at mid-afternoon, and at the end of the working day) to restore stiff, sore, tired, and strained neck, shoulders and wrists.

Yoga postures can be performed statically or in slow dynamics, but always practice in *vinyāsa*, the yoga method in which the movement is accompanied and is synchronous with the breath (the breath is the guide of the movement and expands it). The movement flows with breath and increases in intensity with breath.

Keep each position for at least 30 s (or 10–15 complete respiratory acts of inhalation + exhalation). Follow the sequence displayed in the images of each figure. It is possible to modify the order of the targeted areas, i.e., working on the chest or twisting and then on the wrists. In case of a lack of adequate break time, select 2–3 *āsana* for each area.

The authors recommend reserving a mental space to practice yoga postures, setting an intention and taking deliberate and linked steps towards achieving it.

The proposed yoga-derived *āsana* are:

- *Grivāsana or Kantasanchalana* (Neck rolls)
- *Pranamāsana* (Prayer Pose)
- *Paschima Namaskarāsana* (Reverse Prayer Pose, hands in back side)
- *Purva Namaskarāsana* (Reverse Prayer Pose, hands in front side)
- *Sama Hasta Simbhāsana* (Standing position, hands in lion pose)
- *Upavistha Hasta Simbhāsana* (Seated with hands in lion pose)
- *Upavistha Anuvittāsana* (Seated Backbend Pose with hand palms pushing the sacrum)
- *Anuvittāsana* (variation of Standing Backbend Pose)
- *Urdhva Mukha Upavistha Anuvittāsana* (Standing Upward-Facing Backbend Pose)
- *Upavistha Ardha Purvottanāsana* (Seated Half-Upward Plank Pose)
- *Utkatāsana* (Chair Pose)
- *Parivrtta Utkatāsana* (Twisting Chair Pose)
- *Eka Pada Parivrtta Utkatāsana* (One-Legged Revolved Chair Pose)
- *Upavistha Parighāsana* (Seated Gate Pose)
- *Upavistha Parivrtta Utkatāsana* (Revolved Chair Pose)
- *Ardha Bhuja Gomukhāsana* (Half Cow Face Pose)
- *Upavistha Jathara Parivartanāsana* (Seated Belly Twist A)
- *Upavistha Ardha Matsyendrāsana* (Seated Half Lord of the Fishes A)
- *Upavistha Uttanāsana* (Seated Forward Bending)
- *Dwikonāsana* (Standing Seal Pose)
- *Adho Mukha Svanāsana* (Downward-Facing Dog)
- *Sama Adho Mukha Svanāsana* (Standing Downward-Facing Dog)
- *Sama Urdhva Mukha Svanāsana* (Standing Upward-Facing Dog)
- *Sama Uttana Shishosana* (Standing Extended Puppy Pose)
- *Upavistha Chakrāsana or Urdhva Dhanurāsana* (Seated Full-Wheel Pose or Upward Bow Pose)
- *Sama Tiryaka Bhujangāsana* (Standing Twisted Cobra)
- *Upavistha Meru Wakrāsana* (Seated Twist)
- *Upavistha Sarpāsana or Salabhāsana* (Standing Snake Pose or Locust Pose)
- *Sama Tiryaka Bhujangāsana* (Standing Twisting Cobra Pose)
- *Kati Chakrāsana* (Waist Twist Pose)
- *Trikonāsana* (Triangle Pose)
- *Parivrtta Trikonāsana* (Twisted Triangle Pose)
- *Upavistha Parivrtta Utkatāsana* (Seated Twisted Chair Pose)

Hands gestures (*Hasta Mudras*): *Anjali Mudra* (prayer pose), *Brahma Mudra* (closed fist with thumbs inwards and knuckles pressing each other), *Ganesha Mudra* (locked fingers with palms together rotated in opposite directions), *Ksepana Mudra* (prayer pose with crossed fingers and straight index), *Linga Mudra* (hands together with interlocked fingers, knuckles pointing out, and thumb upright), and *Karkata Mudra* (hands together with entwining fingers and thumbs out).

*Āsana* names were mainly derived from Hathayoga Pradipīkā (XV century) then elaborate and represented iconographically by Tirumalai Krishnamacharya (1934), Bellur Krishnamachar Sundararaja *Iyengar* (1976) and Satyananda Saraswati (1969).

The foundations of the *āsana* technique reside in *B.S.K. Iyengar Yoga*, *Parināma Yoga* and *T. Krishnamacharya vinyāsa Yoga* methods.

## 3. Results

### 3.1. Wrists/Hands

**Figure 1:**
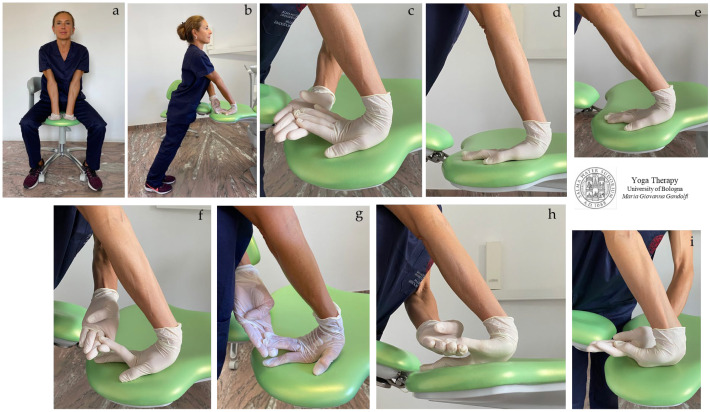
The figure shows *āsana* for stiff and strained muscles of the wrists, palms, and fingers, especially for flexor muscles. *Upavistha Hasta Simbhāsana* (seated with hands in lion pose) (**a**) and *Sama Hasta Simbhāsana* (standing position, hands in lion pose) (**b**–**h**). Muscle lengthening increases markedly when the fingers are stretched upwards, reducing the wrist angle (back of the hand to forearm angle); see images (**d**) vs. (**e**) and (**h**) vs. (**i**).

In Figure 1a–i are shown *āsana* suggested to decompress and reduce the stiffness of wrists and palms. The proposed *āsana* are *Upavistha Hasta Simbhāsana* (seated with hands in lion pose) (Figure 1a) and *Sama Hasta Simbhāsana* (standing position, hands in lion pose, i.e., palms pressing down on the surface with fingers straight backwards) (Figure 1b–i).

The illustrated *āsana* act on the strained and tight flexor muscles of the fingers and thumb, such as *flexor pollicis* and *abductor pollicis,* also on *brachioradialis*, *flexor carpi radialis,* and *flexor digitorum superficialis* when all the fingers are stretched upwards and *palmaris longus* and *flexor carpi ulnaris* when the ring finger and little finger are stretched upwards.

Keep in consideration that the more the palm (carpal and metacarpal area) is lifted up from the plane, the more the stretch shifts from the wrist–forearm to the palm–fingers, and also that the elongation increases in intensity by reducing the angle between hand and forearm. Note that the Figure 1h,i show a movement leading to the enlargement of the narrow passageway forming the carpal tunnel.

### 3.2. Neck

**Figure 2:**
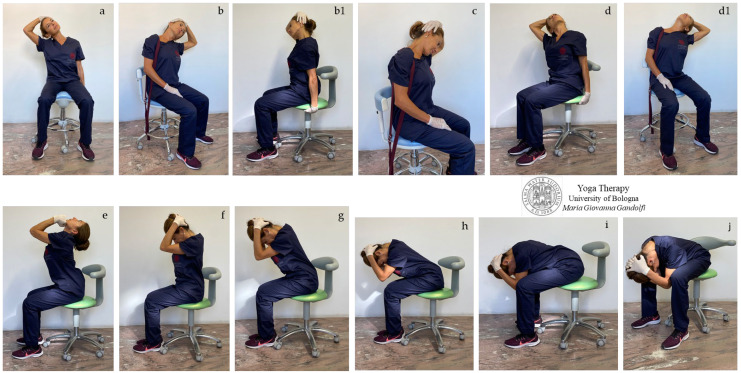
The figure shows *Grivāsana* or *Kantasanchalana* (neck rolls) with lateral bending of the head (**a**,**b**,**b1**), lateral bending of the head with slight axial rotation and flexion (**c**), and lateral bending with slight axial rotation and extension (**d**,**d1**). Keep in consideration that, with increasing head bending, the stretching of the *scalene* and *sternocleidomastoid* (**a**–**e**), *levator scapula* (**c**), *trapezius* and *suboccipital* (**c**,**f**–**j**) muscles, *interspinales,* and *erector spinae* (**f**–**j**) muscles increases in intensity.

**Figure 3:**
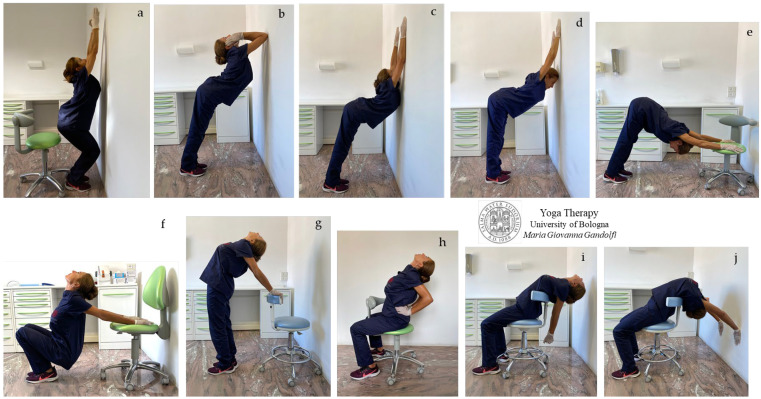
The figure displays the proposed *āsana* for neck and chest extension. *Utkatāsana* (**a**), *Utthana/Sama Hasta Hanu Shishosana* (standing puppy pose with hands on the chin) (**b**), *Utthana/Sama Uttana Shishosana* (standing extended puppy pose) (**c**), *Utthana/Sama Adho Mukha Svanāsana* (standing downward-facing dog) (**d**), *Adho Mukha Svanāsana* (downward-facing dog) (**e**), *Upavistha Ardha Purvottanāsana* (seated half-upward plank pose) (**f**,**i**), *Anuvittāsana* (variation of standing backbend pose) or *Upavistha Sarpāsana* or *Salabhāsana* (standing snake or locust pose) (**g**), *Upavistha Anuvittāsana* (seated backbend pose with palms pushing the sacrum) (**h**), and *Upavistha Chakrāsana* or *Urdhva Dhanurāsana* (seated full-wheel pose or upward bow pose) (**i**,**j**).

In Figure 2a–j and Figure 3a–j are displayed *āsana* involving the neck and upper back, showing detensioning and compensatory movements. The yoga postures selected for the protocol involve movements of extension (bending backward), flexion (bending forward), and neck extension or flexion with associate axial rotation and lateral bending.

It should be emphasized that the cervical muscles join and pull the cervical vertebrae together by approaching their origin in vertebral bone to their insertion in the overlying vertebra or in the occipital/temporal bone. The concept “muscles are bone ties” has been coined and developed by the yoga-expert author to explain visually (during her lectures on posturology and Yoga Therapy) that musculo-tendon structures are bonds/connections pulling together and nearing the bone segments where they are anchored.

Among the eleven muscles connecting head–neck–upper back, *rectus capitis* (inferior nuchal line to C2), *splenius capitis* (temporal bone to C7-T3), *semispinalis colli* (T1–T6 to C2–C5), *splenius capitis* (ligamentum nuchae and C7-T3 to temporal bone), *splenius cervicis* (ligamentum nuchae and T3–T6 to C1–C3), and *longissimus capitis* (C4-T4 to temporal bone) anchor skull bones to the neck and are the mainly involved in forward head support. These muscles, together with *spinalis colli*, *obliquus capitis*, *semispinalis capitis*, *longissimus cervicis,* and *interspinales cervicis,* jointing C-C or T-C, are responsible for cervical spine compression when stiff and retracted.

In addition, anterolateral neck muscles such as anterior *scalene* (transverse processes C3–C6 to first rib), middle *scalene* (transverse processes C2–C7 to first rib), posterior *scalene* (transverse processes C5–C7 to second rib), *subclavius muscle* (first rib to clavicle) and *sternocleidomastoid* muscle (manubrium and clavicle to occipital bone) can compress costoclavicular space and subpectoral space, the subclavian artery and vein, and the nerves of brachial plexus.

*Grivāsana* or *Kantasanchalana,* i.e., the neck movements displayed in Figure 2a–j, show an intense detensioning of the head–neck craniovertebral junctions (decompression of the occipital-atlanto, atlanto-axial, and intervertebral joints) for treatment and prevention of musculo-ligamentous retractions. A deep neck extension having an intense elongation effect on the *sternocleidomastoid*, *scalene* (Figure 2a–e), *levator scapula* (Figure 2c), *trapezius* and *suboccipital* (Figure 2c,f–j) muscles, *interspinales* and *erector spinae* (Figure 2f–j), *longus colli*, *platysma*, *digastric,* and *hyoid* (Figure 2e) muscles is displayed. We highlight that the stretching of the muscles increases in intensity with increasing flexion and bending and also by bending with hands gripping the chair (note Figure 1b vs. Figure 1b1 and Figure 1c vs. Figure 1d). We recommend intensifying the position by following a steady rhythmic deep breathing (no fragmented breath, no apnea). 

Note in Figure 2b,b1,d,d1 the different elongations induced by the arm traction with the arm resting on the leg or with hands gripping the chair.

In Figure 3a–j are shown *āsana* for neck and chest extension; a synchronous hyperflexion of shoulders is practiced in Figure 3c–e,j; and a synchronous shoulders hyperextension is displayed in Figure 3f,g,i. 

The *āsana* in Figure 3f–i show an intense stretch of shoulder muscles, such as *deltoid*, *coracobrachialis*, *biceps*, and also *pectoralis*. In Figure 3c–e,h,j, note the lengthening of the chest muscles, such as the *subclavius* (first rib to clavicle), *pectoralis*, *serratus anterior*, *internal* and *external intercostal,* and *subcostal muscles*, and also *latissimus dorsi* and *subscapularis* and *teres minor* (both rotator cuff muscles) (Figure 3c–e,j). 

The *āsana* in Figure 3f,g display the mobilization of the sterno-clavicular joint, manubrio-sternal joint, and rib attachment areas (costosternal and costocondral junctions) with the intense elongation of the *sternocleidomastoid* and *subclavius* muscles, useful for the prevention of upward dislocation of the clavicle and compressive syndromes.

We teach going deep into the *āsana* with a concentrated mind, the breath driving, and rhythmic body movement. We recommend training stillness into uneasy *āsana* when breathing is difficult. Learn to find calm in discomfort. 

We would introduce the concept that tensegrity in *Yógāsana* implicates that each movement (even if small) pulls, stretches, and moves muscles, ligaments, and organs through pervasive fascia. 

Therefore, we solicit to turn your attention inward to the internal structures of throat and chest, to search for tension and movement perception.

Focus on the thoracic extension, the arched back. Move the sternum and throat outward using the power of the breath.

We need to highlight that the extension of the thoracic spine, its mobilization, and the removal of restrictions is crucial for the physiological (healthy) functioning of the abdominal organs (strongly compressed by hyperkyphosis or a depressed chest or rounded back) and diaphragm movement. *Āsana* in Figure 3c,f,g,i and mainly j, reactivate and rehabilitate diaphragmatic breathing favoring venous return and lymphatic drainage, and parasympathetic nervous system activation with beneficial effects on cardiovascular system. 

Dorsal hyperkyphosis favors reduction in the mobility of the sterno-clavicular joint (the mobility of a healthy joint being high, with 35° horizontal, 70° anteroposterior, and 45° rotary movement) and the posterior displacement/dislocation of the sterno-clavicular joint towards a depressed position.

We would elucidate that thoracic extension is the ability for the t-spine to move from its normally kyphotic or forward rounded position to a flat or arched back position. The lack of thoracic extension is a very common mobility restriction. At the beginning of the mobilization, pain and discomfort can occur and can be disquieting and even strong as the body resists. By surrendering to it, we soften the body, and gradually it will lessen. Concentration on the breath leads to softening of the body to lessen the discomfort and a pleasant muscular and mental surrender.

### 3.3. Shoulder Girdle

**Figure 4:**
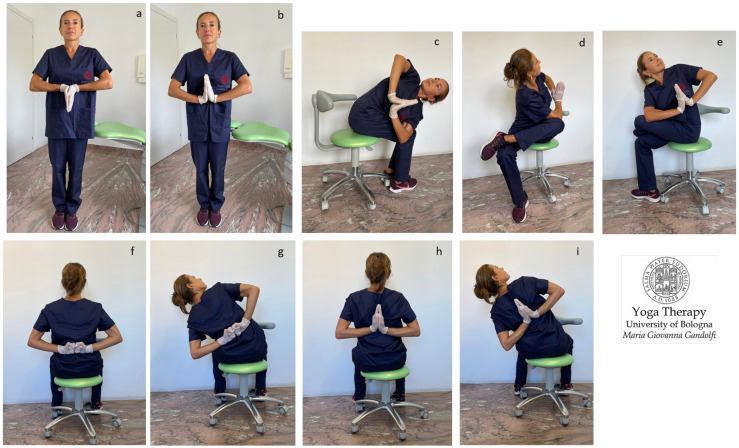
The figure shows the proposed *āsana* for shoulder mobilization. *Purva Viparita Namaskarāsana* (**a**), *Purva Namaskarāsana* (prayer pose, hands in front side) (**b**), *Purva Namaskarāsana Parivrtta Utkatāsana* (twisting chair pose with hands in prayer pose in front side) (**c**), *Eka Pada Parivrtta Utkatāsana* (one-legged revolved chair pose) (**d**,**e**), *Upavistha Paschima Brahma Mudra* (**f**,**g**), *Upavistha Paschima Namaskarāsana* (**h**) and *Parsva Paschima Namaskarāsana* (side bending with hands in prayer pose on back front) (**i**). The stretching and destressing the of *infraspinatus* and *teres* (mainly *teres minor*) rotator cuff muscles is intense (**f**–**i**).

**Figure 5:**
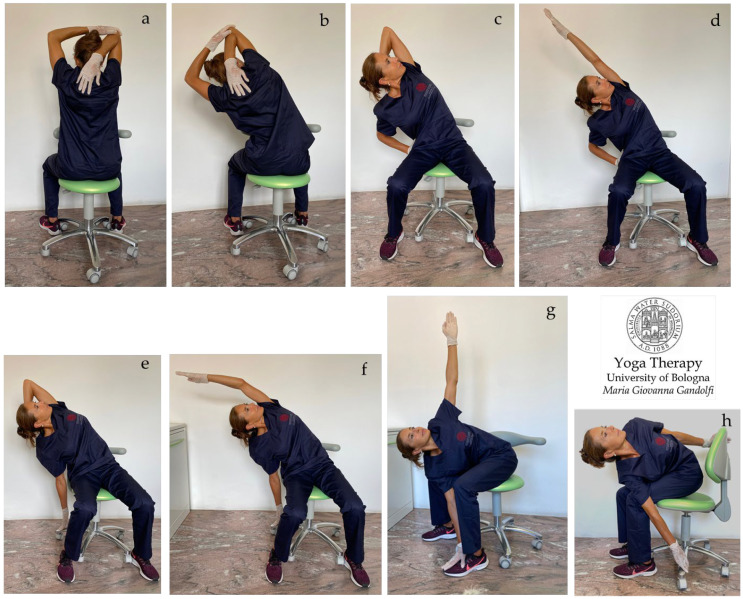
This figure displays the proposed *āsana* for the shoulder, chest, and thoracic spine. *Ardha Bhuja Gomukhāsana* (**a**,**b**), *Upavistha Ardhachandrāsana* (**c**–**f**), *Parivrtta Utkatāsana* (**g**), and *Parivrtta Baddha Utkatāsana* (**h**).

**Figure 6:**
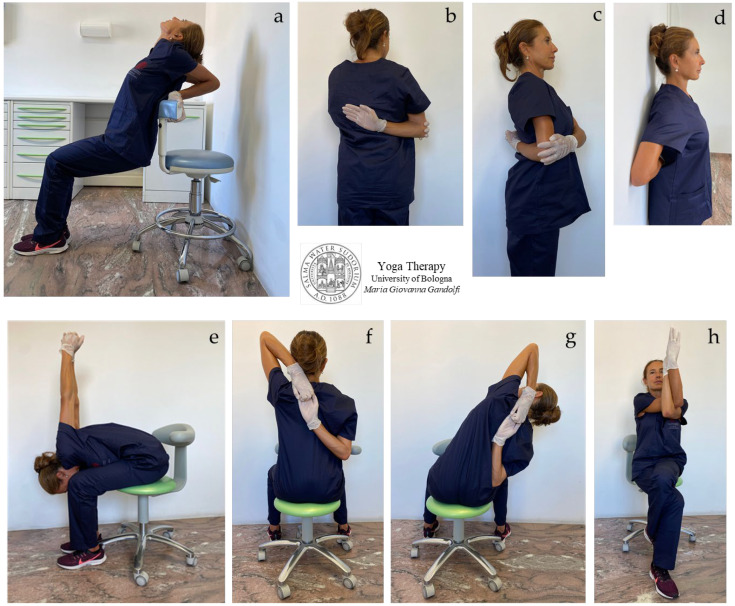
The figure illustrates *Yógāsana* proposed for shoulder and chest mobilization and elongation. Arms in *Sarvangāsana* (**a**), preparation of shoulders for *Garudāsana* (**b**–**d**), *Garudāsana* (eagle pose) (**h**), *Bhuja/Hasta Garudāsana* (arms/hands in eagle pose), then with side bending (**f**,**g**), and *Upavistha Prasarita Padottanāsana* (**e**).

In Figure 4a–i, Figure 5a–h and Figure 6a–h are shown *āsana* involving the upper body (shoulder, chest, and thoracic spine).

We illustrate different *āsana* working also on stiff and strained wrists and palms (Figure 4a–e,h,i) and for tight shoulders (Figure 4f–i). The illustrated *āsana* act on tight flexor muscles such as *flexor digitorum*, *flexor carpi radialis*, *palmaris longus,* and *flexor carpi ulnaris*. Figure 4a,b shows the *Anjali Mudra* to mobilize and loosen wrists in preparation for subsequent movements. Figure 4f,g displays the *Brahma Mudra* as an intermediate movement to prepare for the next one (Figure 4h,i).

The shoulder movements of the protocol include the medial internal rotation of the humerus in adduction, with hands in front of (Figure 4b–e) or behind (Figure 4f–i) the torso. In these movements, the scapulohumeral rhythm is restored, as the scapula is solicited into substantive three-dimensional motions, namely upward/lateral rotation, protraction/internal rotation, and anterior tilt with an associated stretch of *serratus anterior* and the *trapezius*, *rhomboids*, *levator scapulae*, *deltoids*, *infraspinatus,* and *teres* rotator cuff muscles.

Figure 5a–h displays side bending and twisting of the body trunk to stretch the body sides and destress the intervertebral muscles (*intertransversarii* and *interspinales*) and *erector spinae*. Figure 5a,b also shows movements for the shoulders. Figure 5a–h illustrates *āsana* to stretch and loosen the shoulders and trunk side, with elongation of the *triceps*, *serratus posterior*, *latissimus dorsi*, *rhomboids,* and *quadratus lumborum* muscles.

We recommend taking a deep slow breath and then entering into the *āsana*. Remain for some breaths in the position and then (if the body allows) intensify the movement during each expiration with a focused mind and the breath driving body bending or twisting. Avoid haste, apneas, and judgments.

Figure 6a–h shows *āsana* for the chest, shoulders, and trunk side, with elongation of the *pectoralis*, *subclavius*, *serratus anterior*, *intercostales* (Figure 6a,e), *triceps* (Figure 6f,g)*, rhomboids*, *trapezius*, and *levator scapulae* (Figure 6b–d,h), and *latissimus dorsi* (Figure 6e) muscles. 

We propose the concept that scapulohumeral or scapulothoracic kinematics involve synchronous, combined, three-dimensional movements at the sternoclavicular joint, acromioclavicular joint, scapulothoracic joint, and glenohumeral joint. The freedom of movement of all these joints and related bony structures prevents shoulder pain and various disorders such as shoulder impingement syndrome. The shown movements can provide relief for shoulder impingement (Figure 6a–h) and thoracic outlet syndrome (Figure 6a,d,e). In particular, the movements shown in Figure 6a,e can reduce the compression and impingement of the subclavian artery and vein and brachial plexus nerves. The *āsana* performed in Figure 6h strongly extend the *trapezius* (occipital bone, ligamentum nuchae, spinous processes T1–T12 to the clavicle, acromion, and scapular spine) and *levator scapulae* (transverse processes C1–C4 to the scapula medial border). The *āsana* in Figure 6b–d,f–h also heavily lengthen *rhomboid major* (spinous processes T2–T5 to the scapula medial border) and *rhomboid minor* (nuchal ligament, spinous processes C7-T1 to the scapula medial border).

### 3.4. Spine Twist and Side Bending

**Figure 7:**
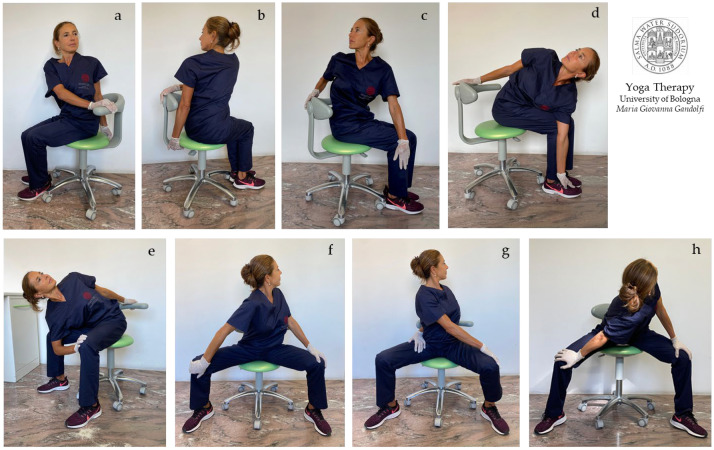
The figure shows *āsana* for the spine and shoulders. *Upavistha Parivrtta Utkatāsana* (**a**–**d**) and *Parivrtta Upavistha Utkata Konāsana* (seated twisted goddess pose) (**e**–**h**). Visualize the stretch in three directions. Awareness is required to practice the displayed *āsana* following breath-rhythmed and guided movements. Handle tension via deep inflation/inhalation and relaxing exhalation.

Figure 7a–h shows *āsana* involving thoracic spine twisting and side elongation. Stretch away your body with an inwardly directed mind.

We would note the biotensegrity, i.e., the principle of the ubiquitousness of tensegrity in all biological structures formed by an interconnected fascial network that distributes forces within itself. Tensegrity can be visualized as floating compression, a balance between space and tension, a compression inside a net of continuous tension where the components must not touch each other.

We would highlight that the thoracic spine should have freedom of movement in flexion (Figure 6e,g), extension (Figure 6a), and rotation (Figure 7a–h), with a higher range of movement in flexion. Rotation of the thoracic spine is responsible for about 80% of trunk rotation. The lumbar spine, as well, is involved in flexion and in the rotation movements displayed in Figure 6e,g and Figure 7a–h, with a lower range of motion compared to the thoracic and cervical spine.

### 3.5. Side Bending

**Figure 8:**
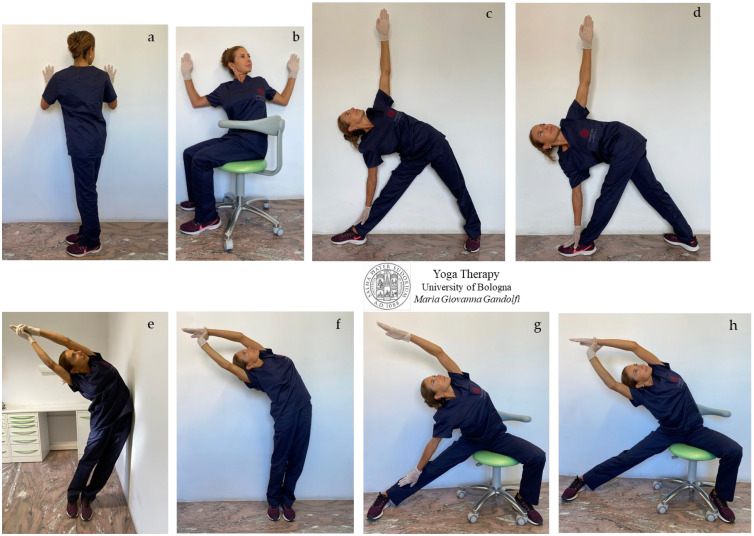
The figure displays *āsana* proposed for a strained and stiff spine. *Kati Chakrāsana* (waist twist pose) or *Sama Tiryaka Bhujangāsana* (**a**), *Upavistha Parivrtta Utkatāsana* (**b**), *Trikonāsana* (**c**), *Parivrtta Trikonāsana* (**d**), *Chandrāsana* (**e**,**f**), *Upavistha Parighāsana* or *Nrtyamaha Virabhadrāsana* (**g**,**h**).

Figure 8a–h shows twisting (Figure 8a,b,d) and side bending (Figure 8c,e–h) *āsana* for the spine.

A three-dimensional active stretch has to be applied to loosen and to engender both elongation and space inside the compressed structures by means of pushing muscles.

Drive your bones away from each other while the focus dives inside with every breath (inhalation).

Always keep in mind the concept of biotensegrity, considering that each movement involves different (even distant) areas through *fascia*.

**Figure 9:**
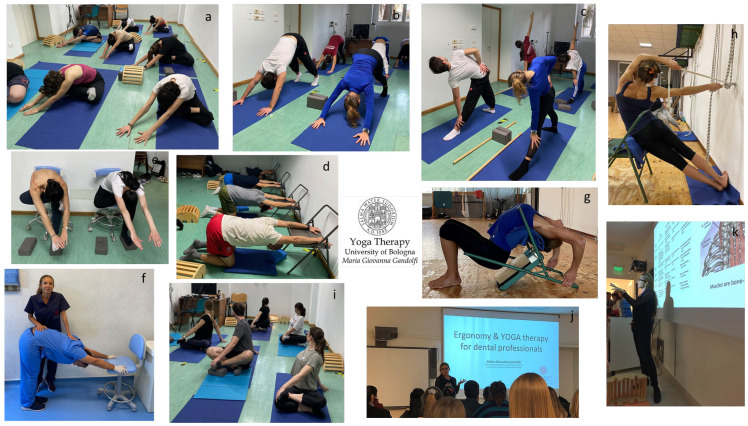
The figure shows *āsana* for the hips (**a**,**c**,**e**,**h**,**i**), shoulders (**b**,**d**,**f**,**g**), and back of the body (**b**,**d**,**f**,**g**) and trunk (**a**,**c**,**e**,**h**,**i**). Future dentists, dental hygienists, and medical doctors specializing in sports medicine practicing *Parsva Parvatyāsana* (**a**); *Adho Mukha Svanāsana* (**b**,**d**,**f**); *Trikonāsana* (**c**); *Parsva Eka Pada Utkatāsana* (**e**); intense thoracic spine extension *Urdhva Upavistha Uttana Shishosana* (**g**) and *Chandrāsana* lateral flexion (**h**) performed following the *Parināma Yoga* technique; *Parivrtta Samidha Sthapanāsana* (**i**). Theoretical Yoga Therapy lesson (**j**,**k**) for students of the dentistry degree or the dental hygiene degree and for medical doctors specializing in sports medicine.

Figure 9 shows students practicing Yoga Therapy at the Dental School of our University as an integrative holistic discipline propaedeutic to ergonomics in dentistry (for dentists and dental hygienists) and to sports medicine (for medical doctors specializing in sports medicine). The Yoga Therapy program includes 120 min sessions, incorporating *āsana* and *prānāyāma* practice and also the teaching of exercises for in-office *Yógāsana* practice.

The aim of this innovative university program is to transmit the concept that yoga trains and teaches constant awareness toward mind fluctuations and body conditions, and develops focused attention, concentration, and body control useful for the self-control and awareness of the posture.

Yoga leads to an open mind, brings curiosity to experiences and serendipity in findings and discoveries, increases sensitivity, and develops spirituality through personal growth, transformation, and turning to the inner dimension. Yoga focuses on a personal journey of self-discovery.

## 4. Discussion

### 4.1. Our Project

This paper represents a yoga-based guideline for the self-cure of musculoskeletal disorders among dental professionals.

The project of the present study was born from our experience of over 10 years of university teaching of ergonomics to the students of the dental school, and from the perceived need for dental professionals to find relief (possibly a non-drug solution) to their work-related pain and to their sore, strained, and tired bodies. There is a manifest necessity and pressing need to take care of oneself.

The presence of an expert yoga guide/academic to teach and train the technique of correct *āsana* practice could overcome the limits of this written protocol despite its accurate details. To date, a survey study on self-awareness and a clinical study on the effects of our Yoga Therapy program are in progress (ethical committee approval protocol number: 847/2021/OSS/AUSLBO).

Nowadays, ergonomics seems mostly to be a commercialized posturology. The marketing of ergonomic products is mostly powered by a never-ending supply of new devices and furniture with new designs and technology.

Ergonomic guidelines provide theoretical recommendations concerning body position (and movement) and mainly focus on the clinic organization. The compliance with recommendation is frequently unsuitable for working rhythms and required dental maneuvers/activity, and are mostly unsuitable and hardly feasible for the poor self-awareness of the body, lack of contact with the body, and the inability to feel one’s own body position, especially when attention is directed elsewhere.

We summarized the ergonomic recommendations in different occupational fields (Table 1) to describe the adequate working posture for dental professionals.

We highlight that these are theoretical recommendations for body position, rarely followed for several reasons:

- lack of postural awareness/perception;
- lack of knowledge of dental ergonomics guidelines;
- unreliable recommended positions in various clinical maneuvers;
- lack of practical–theoretical teaching in university ergonomics programs for dental professionals;
- lack of exercises training posture improvement.

There is a crucial need to train people to control body processes and movements that normally occur involuntarily and to gain control over unperceived or involuntary activities. There is a great need to educate people in concentrative practices, to develop the embodied mind, to build body–mind connection and integration, and to create a continuous body–mind dialogue. Our project, extended beyond this article, aims to spread the science of yoga through dental professionals. Unexpectedly and fortuitously, we found that recently the Harvard School of Dental Medicine of Harvard University proposed a webinar on yoga, and, similarly the Tufts University of Dental Medicine incorporates the teaching of mind–body practices into the curriculum of dental students.

There is a need for enlightened minds and mental openness to turn to and to embrace new (although millennial) sciences and disciplines.

Back to our study, the proposed Yoga protocol has been specifically conceived for dental professionals (dentists, dental hygienists, and dental assistants) to be practiced on dental stools or using the walls of the dental office or a dental unit chair, and includes both sitting and standing positions, with twisting, flexions, extensions, forward bends, and arching to mobilize, decompress, and provide nutrition and oxygen to the musculo-articular system. Movements in all directions of space have been planned. In the designed protocol, the *āsana* techniques arise from *Hatha Yoga* and from its modifications and elaboration from *Iyengar Yoga* and *Parināma Yoga*.

*Hatha Yoga* practice is structured around focused attention on breathing (*Prānāyāma*) and postures (*āsana*), while a non-judgmental attitude and acceptance of one’s current psycho-physical state is cultivated in open monitoring. *Iyengar Yoga* and *Parināma Yoga* adopted the use of supports, tools and ropes to allow, favor, or increase (depending on the body condition of the yoga practitioner) the correct physiological movement of the articulations.

### 4.2. About Yoga Significance

Yoga trains continuous contact with body, the continuous unaware body–mind dialogue. Yoga develops awareness, being-presence, concentration, and inward attention.

Yoga is not intended for flexible people. Yoga is for people who decide to take care of themselves, looking for a connection with their own body and a way to quiet the mind.

Yoga requires intentional self-discipline, including regular daily exercise (considered as meditation), attention, intention, and disciplined action. Yoga generates serendipity as it cultivates an open, non-judgmental attitude and a sensitive mind. Yoga is the discipline of the mind, senses, and physical body.

Yoga, as integrative science in medicine and in public health, represents the path for holistic well-being.

Yoga can help both in daily life and in business providing calmness of mind, confidence, mental strength, increased energy and restful sleep. Yoga allows mastery over the mind, emotions, and impulses by calming emotional reactions. The practice of yoga makes a person inwardly even-minded.

Yoga is a movement-based contemplative practice in which attention, perception, and intention are attitudes to develop the embodied mind, i.e., the mind and cognition shaped and influenced by the body and its relationship with incoming information. Perception and action become oriented, motivated, and purposeful for exploring qualities of embodiment through yoga practice, affecting the individual whole relationship with the world.

### 4.3. Yoga Benefits

We are aware that most people intend yoga or come to yoga simply as a means to stretch, work out, relax or quiet down. *Yógāsana* is a great form of physical exercise and is primarily a physical discipline. The very immediate effect of practice is a feeling of physical and mental well-being; the body strengthened and the mind quieted and cleared. Yoga practice shifts towards a completely different way or mode of being. Yoga trains and cultivates a number of positive, skillful qualities in the mind, such as concentration and presence; the mind increases awareness, flexibility, and balance. 

Yoga is a form of preventive medicine generating physical well-being. Yoga promotes freedom of movement by stretching tight muscles, loosening hardened joints, and undoing accumulated tension held in the body. It relaxes the body and develops stamina. Interestingly, the U.S. Department of Health and Human Services National Institutes of Health (NIH) National Center for Complementary and Integrative Health has produced and disseminated the eBook Yoga for Health as evidence of the importance of yoga. 

In this discussion, we would highlight that our innovative university Yoga program is focused to train self-control and posture awareness through the development of awareness, focused attention, dialogue with own body, and increase the self-cure. Moreover, experimentation on oneself through *āsana* allows one to became aware and to deal with specific problems, such as neck pain and headaches and stress-related disorders. 

To obtain profound, significant benefits, yoga requires, discipline, commitment, and regular practice with conscious intention; yoga needs to apply effort, will, and determination. We need to come back again and again to our practice, repeating the same movements again and again, seeking to work more effectively, more deeply each time, or simply become aware of the physical conditions of the moment.

#### 4.3.1. Yoga for Stretching Your Zone of Tolerance and Training Patience

Most of us spend the majority of our time within our comfort zone or “zone of tolerance”. This way of being refers to our mental pattern, thoughts, emotions, actions and physical movements. I encourage my students to create tolerable discomfort during the yoga practice, in a way to immerse themselves in discomfort/unfamiliar. 

Yoga practice generates tolerance and endurance (*titiksha*) of discomfort, unfamiliarity or unhappiness; increases forbearance (patient endurance of suffering) and endurance of afflictions without countering aids and without anxiety or lament, by developing non-reactivity, a sort of indifference towards discomfort and hardship.

The practice of yoga makes a person inwardly even-minded and patient, increases mental balance, and calms the emotional reactions.

#### 4.3.2. Yoga for Muscle Retraction Inside the (Bio)Tensegrity Concept

Muscle retraction and stiffening consists of persistent and abnormal reductions in length as a result of the shortening of muscle and tendon fibers, often accompanied by atrophy and degeneration of the affected tissues. Retraction occurs as a consequence of prolonged contraction during clinical activity, leading to strained and stiff (and sore) musculo-tendon structures. Retraction becomes permanent if shortened and stiffed tissues are not regularly outstretched and softened, with subsequent pain and histological alteration of musculo-tendon units.

Neck extensors and the upper back musculature are at a high risk of retraction with the prolonged working postures required by dental practice, mainly with the head forward and trunk bending forward, leading to stable postural alterations.

We introduce the concept that *fascia* is a tensegritous, uninterrupted, viscoelastic matrix of connective tissue (a three-dimensional web of fibrous, gluey, and wet collagen fibers) that surrounds, penetrates, connects, and supports all body structures, from cells to organs and tissues. Fascia has different viscosities, densities, strengths, and resiliencies depending on its usual movements, and the tension of the fascia supports the body. In the musculoskeletal apparatus, the fascial system connects muscles, bones, ligaments, and tendons, creating a tensile and compressive balance that unifies and distributes the force of any movement through the whole body seamlessly. When something moves, everything moves. Living bodies are biotensegritous organisms. Biotensegrity indicates the tensegrity (or tensional integrity or floating compression) in all biological structures and describes the presence of tensioned and compressed parts. Biotensegrity implies that movement of the musculoskeletal apparatus will create tension or compression in every body structure.

For these reasons, it is very important to practice *āsana* with intentional three-dimensional stretching away, and to consider that every deep, intense elongation moves and activates (close or distant) organs, glands, and the nervous system.

### 4.4. Work-Related Musculoskeletal Disorders in Dentists

Dental professionals are exposed to many different occupational hazards during the course of their professional activity, such as physical, chemical, biological, and ergonomic factors. Ergonomic hazards, caused by strained postures and prolonged repetitive movements, trigger musculoskeletal disorders (MSDs). Currently, work-related musculoskeletal disorders are a major health problem among dental professionals, showing a prevalence ranging from 64% to 93% [1,2,3,32], in agreement with our recent study showing 85% prevalence [3].

The literature reports that the risk of musculoskeletal disorders among dental professionals also varies according to the prevalent dental activity. In a survey-based study, a cohort of dentists (*n* = 525) highlighted a higher risk of MSDs for periodontists (90.6%) and endodontists (92.6%) [33].

The working posture of dental professionals has been identified as a major risk factor for the development of work-related MSDs.

A recent systematic review analyzed ergonomic risk factors and preventive measures for musculoskeletal disorders in dental practice, in 29 articles and reported that the main risk factor for the development of MSDs is the prolonged static posture (highlighted in 87.5% of reviews and 84% of original articles) followed by repetitive movements, muscle imbalances, sedentary life, and obesity. The review also highlighted the importance of stretching after each working session and at the end of the working day [8].

Another recent review analyzed 30 studies and reported that the neck (58.5%) and shoulders (43.1%) are the most affected by musculoskeletal diseases and pain among dental professionals and highlighted that working posture is the major risk factor for pain and MSDs development [2].

The authors of the present paper wish to emphasize that dentistry is a physically demanding profession which obliges prolonged static postures with the head forward, cervical flexion and rotation, forward bending of the upper body or forward bent trunk posture, torsion and inclination of the neck and trunk, abducted arms, prolonged flexion of the arms and forearms, constant activation of the finger flexors, high muscular strain, repetitive precision-demanding handgrips, etc.

The most frequent occupational cervico-brachial disorders and pathologies among dental professionals include [3] (acute or chronic) pain, tension and migraine headaches, compressive syndromes, compressive neuropathies, carpal tunnel syndrome, De Quervain tenosynovitis, pronator syndrome, lateral epicondylitis, tension neck syndromes, (neurogenic/arterial/venous) thoracic outlet syndrome, impingement syndromes, subacromial impingement, and shoulder disorders.

The etiopathogenesis of musculoskeletal disorders is related to muscle injury for fatigue, imbalance, and microtrauma (mainly for extreme or unbalanced posture and repetitive movements) allowing to trigger points, compressions, and muscle ischemia/necrosis. At a cellular level, mechanical injury to muscle fibers and articular tissues causes cell disruption with diffusion of intracellular components and cellular release of cytokines and proinflammatory mediators that activate inflammatory cells direct to tissue damage followed by pain and inflammation. Pain causes protective muscle contraction, joint hypomobility, and nerve compression, with consequent cumulative trauma disorders and establishment of a vicious circle (Gandolfi, unpublished data) [3].

### 4.5. Yoga vs. Pain

An extensive meta-analysis reported the usefulness of yoga exercises for pain and several pain-associated disorders. Moreover, there is evidence that even short-term yoga interventions might be effective [34].

Yoga can recondition and heal the neuromuscular system by improving blood rush, promoting the nourishment of muscles and other musculoskeletal tissues (fascia, tendons, ligaments, etc.), by lubricating the joints, strengthening periarticular muscles, and improving the coordination of joint movements [3].

A recent cross-sectional survey of Australian dental hygienists (*n* = 85) reported the use of several complementary treatments (including yoga-based exercises, not specifying the type of yoga) to reduce pain and disabilities provoked by MSDs [35].

A clinical prospective study on young patients (*n* = 106) reported that *Iyengar Yoga*-based exercises (stretching and relaxation) were effective in reducing chronic pain after rehabilitation [36].

### 4.6. Yoga-Like Exercises in Dental Office

Only poor articles have been published on yoga-derived exercises to be performed in dental offices. Just a few movements have been proposed, with superficial and incomplete technique descriptions (and unreported *āsana* names) or benefit explanations, namely neck rolls (*Grivāsana*), shoulder rolls (*Skandhāsana*), forward bending (*Uttanāsana*), *Urdhva Hastāsana*, *Anuvittāsana* (extension of body front), *Upavistha Salabhāsana*, seated forward bend (*Upavistha Uttanāsana*), *Upavistha Urdhva Hastāsana*, prayer pose (*Purva Namaskarāsan*), and shoulder stretch (*Eka Bhuja Padmāsana*) [37,38,39,40].

In our paper, we innovatively propose a Yoga protocol displaying *āsana* and movements to be performed daily in the dental office. We provide a tool for the prevention or treatment of musculoskeletal disorders among dental professionals, with the purpose to spread yoga as medical science for physical and psychological well-being.

### 4.7. Carpal Tunnel Syndrome in Dentistry

The carpal tunnel is a narrow passageway located on the palms of the hands—enclosed between the carpal bones and the transverse carpal ligament—constituting the passage of the median nerve and flexor tendons. The transverse carpal ligament acts as an anchor for the three thenar muscles, namely the abductor pollicis brevis, flexor pollicis brevis, and opponents pollicis.

When the median nerve is compressed, symptoms can include numbness, tingling, and weakness in the hand and arm.

A clinical study from the University of Michigan published on JADA diagnosed a median mononeuropathy in 13% of 1079 dentists [41]. In a large study of American dental hygienists (*n* = 177), the prevalence of self-reported carpal tunnel syndrome symptoms was almost 56% [42].

Studies indicate that 6.4% to 11% of all dental hygienists are diagnosed with carpal tunnel syndrome [43,44].

#### Yoga vs. Carpal Tunnel

A clinical study from the University of Philadelphia published on JAMA analyzed the effectiveness of Yoga Therapy in 42 patients affected by carpal tunnel syndrome randomly divided in three treatment groups. The treatment group received an *Iyengar Yoga*-based intervention consisting of 11 yoga postures designed for strengthening, stretching, and balancing each joint in the upper body along with relaxation for a 1.5 h session twice per week for 8 weeks. The control group received a wrist splint. The yoga group had a significant improvement in grip strength and pain reduction. Yoga practice was significantly more effective on the wrist flexion test than wrist splinting or no treatment in relieving some symptoms and signs of carpal tunnel syndrome, although non-significant in the tingling-to-percussion test [45].

A commentary on the above-mentioned study from the University of Chicago published in The Lancet suggested the use of yoga *āsana* (prayer pose and *Tadāsana*) to restore stiff and strained/retracted musculoskeletal structures to prevent carpal tunnel syndrome, suggesting surgery only in patients unresponsive to conservative regimens [46].

In our proposed Yoga protocol for carpal tunnel, we propose different *āsana* for decompressing and reducing the stiffness of wrists and palms, as shown in Figure 1a–i and Figure 4a–e,h,i. The proposed *āsana* act on the strained flexor muscles of the fingers and thumb flexor muscles, and also on the brachioradialis and flexor carpi radialis when thumb and index are stretched upwards; acts on the flexor digitorum superficialis when all fingers are stretched upwards, and on palmaris longus and flexor carpi ulnaris when ring finger and little finger are stretched upwards.

### 4.8. Neck Disorders among Dentists

The literature reports that work-related MSDs in the neck and shoulders represent an important problem in occupational medicine. Our recent survey study reported that 59.9% of dentists and dental hygienists suffer from neck and shoulder pain [3].

An extensive review reported the risk factors for and prevalence of neck and shoulder disorders among dental practitioners (dentists, dental hygienists, and dental assistants). Neck disorders/symptoms affected 26–73% of dentists, 54–83% of dental hygienists, and 38–62% of dental assistants [4].

A recent survey-based study reported that the prevalence of neck pain was significantly higher in dentists (*n* = 100) (73%) compared with office workers (controls, *n* = 102) (52%) [5].

A Scandinavian survey-based study on dentists (*n* = 99) and pharmacists (*n* = 100) reported that 44% of dentists suffered from neck symptoms, 51% from shoulder symptoms, and 12% from forearm symptoms, while a significantly lower percentage was found in pharmacists. Upper limb numbness and paresthesia were more common among the dentists [47].

A recent survey-based study on the prevalence of MSDs occurring in the last 12 months among dentists (*n* = 450) reported a prevalence of 65.6% during the last 7 days, 92% during the last 12 months, and 95.8% in their lifetime. The most-affected body regions were the neck (42.7%–70.9%–78.4%) and shoulders (29.8%–55.6%–66.2%) [7]. 

Interestingly, dental students also resulted highly affected by cervical neck disfunctions [48,49]. A survey-based study in one university department on dental students (*n* = 112) reported a high prevalence of cervical spine disorders (54%), chronic neck pain (57%), headache (28%), stress (58%), and neck movement disabilities (55%) [48]. A longitudinal survey-based study on dental students (*n* = 73) of a university dental school reported that 61% had neck complaints, increasing between the first and the fifth year of practice [49].

We want to illustrate the concept that, when muscles are tense, they shorten. Shortened muscles put the involved articulation into compression. Shortened muscles force many other different muscles to overwork, to perform a movement or to keep the postural balance. Once the muscles become painful, a vicious cycle begins. The pain increases the tension, the soreness, the unbalanced movement, and the strain. This worsens the muscle spasm, which in turn increases the pain.

#### 4.8.1. Forward Head Posture 

We will explain the concept that the working position of dental professionals obliges to a forward head posture that means the head is misaligned with the cervical spine, protruding (forward) away from its center of gravity. A forward head posture is defined by forward translation of the cervical vertebrae with overextension of the muscles of the neck front, and tightness and stiffens of the muscles of the back. Head protrusion is a major cause of (tension) headaches in relation to the stress and effort required to sustain the head misaligned to the cervical spine.

In our ergonomics and posturology teachings, we indicate that the head should be balanced directly above the spine, so that the earlobe is in line with shoulder acromion. 

We would like to state that the weight of an adult human head is approximately 5.44 kg (12 lbs.) when aligned on top of the spine. The weight of the head will increase by 4.54 kg (10 lbs.) for every 2.54 cm (1 inch) displacement forward in the sagittal plane away from its center of gravity [50].

Therefore, an apparently insignificant small forward displacement of 7–8 cm (3 inches) during dental practice increases the head weight to 18 kg (40 lbs). The head weight further increases from 18 kg when flexed at 30 degrees up to 27 kg when protruded 60 degrees [50], with a consequent strong load weight on the cervical region and neck muscles obliged to isometrically restrain tens of kilograms against the unrelenting force of gravity. This increases tremendously the level of stress on both the lower cervical and upper thoracic spine as the upper back and neck muscles must work much harder to stabilize and support the head. That stress often leads to neck pain, shoulder pain and muscle tightness, (tension-derived) headaches—irradiating from the base of the skull (occiput) to the front (tension headache) or nape (hypertension headache), or to the eye-cheek (migraine)—and likely cervical disc degeneration.

#### 4.8.2. Headache among Dentists

An estimated 80% of all headaches occur from muscle tension.

We want to elucidate that dental professionals are at high risk for muscle-tension headache as head protrusion is a major cause of tension headaches. Stiff, strained, and retracted neck muscles from postural imbalance affect head pain by their direct anchoring to the bones of the skull, and the pain from tight neck muscles can spread to other areas of the body.

A survey-based study from the University of Sidney highlighted that headaches represent a frequent symptom affecting dental professionals. It has been reported that 58% of dentists (*n* = 355) experienced headache in the previous month and 25% of them suffered severe symptoms [51]. Headaches were often associated with neck and shoulder symptoms.

A more recent joint survey-based study from the universities of Harvard and Michigan reported prevalence of 17.8% for headaches and 46.6% for neck pain in dentists (*n* = 399) [52].

#### 4.8.3. Yoga vs. Disk Herniation and Bulging 

A recent pilot study from a Turkish University analyzed yoga-based interventions in patients with neuropathic pain due to disc herniation (*n* = 48). One group was randomly selected to perform for one hour twice weekly for 12 weeks a Yoga program including 16 different listed *āsana* (unspecified yoga style), while the other group was assigned to control [28]. The results of the study indicated that the selected yoga exercises reduced neuropathic pain, lower back pain, and disability, being a promising treatment option in these patients [28].

In a randomized controlled trial from Birmingham University, patients with disc extrusions or bulges (*n* = 61) were randomly assigned to 30 min daily yoga exercises at home (yoga style not specified) after 3 months or to no intervention (control). People with extruded discs benefited from Yoga Therapy in both sciatica and nonspecific back pain, with improved pain-related and self-reported disability scores [52].

In a nationwide retrospective study from a Chinese University, dentists (*n* = 10,930) were compared to an age-matched general population (*n* = 73,718) in order to analyze the incidence rate of cervical herniated intervertebral disc. The study found that younger dentists (under 34 years) had almost twice the risk of cervical herniated intervertebral disc development than the younger general population [53].

In a clinical case control study from a Chinese University, long-term yoga instructors (yoga style not specified) (*n* = 18) were compared to non-yoga-practicing individuals (*n* = 18) to assess the presence of cervical and lumbar disc degenerative disease. Magnetic resonance imaging showed that long-term yoga instructors had significantly less degenerative disc disease than non-yoga-practicing individuals [26].

In another study from the University of Geneva, patients with a diagnosis of neck herniated discs, shoulder pain, or radiating arm symptoms (*n* = 30) underwent spinal therapeutic exercises consisting of 10–15 times each session, 6–10 sessions per day for 8 weeks. The 8-week therapeutic exercises led to significant changes in the intervertebral foramen areas. In particular, the study showed that active cervical flexion increased the area of the patients intervertebral foramen from C2–C3 to C6–C7, and the extension exercise reduced the foramen area from C2–C3 to C6–C7. As consequence, pain and the neck disability index of these patients significantly decreased after the therapeutic exercise intervention [54].

A recent review analyzed the effectiveness of *Iyengar Yoga* in treating neck and back pain. The review included a total of six studies and offered strong evidence for the short-term effectiveness but little evidence for the long-term effectiveness of *Iyengar Yoga* for chronic spine pain on patient-centered outcomes [55].

Yoga is often recommended as an evidence-based additional therapy intervention for back and neck pain. Being more than exercise, yoga improves body awareness, pain acceptance, and coping. The review identified some *āsana* designated to reduce spinal pain, namely *Tadāsana*, *Ardha Uttanāsana*, *Chair Bharadvajāsana*, *Adho Mukha Virāsana*, *Adho Mukha Svanāsana*, *Utthita Trikonāsana*, *Virabhadāsana II*, *Utthita Parsvakonāsana*, *Prasarita Padottanāsana*, *Supta Padangustāsana*, *Nyubja Savāsana*, *Supta Pavanamuktāsana*, and *Supta Savāsana* [55].

#### 4.8.4. Yoga vs. Neck Pain

A review from the University of Berlin was made to systematically assess the effectiveness of yoga in relieving chronic neck pain. The review, performed by a research group highly prolific in yoga studies (*n* = 68 studies), highlighted the short-term effects of yoga on chronic neck pain, its related disability, quality of life, and mood, suggesting that yoga *(Iyengar Yoga*, *Ashtanga Yoga*, and *Kriya Yoga*) represents a good treatment option [56].

A randomized trial from the University of Berlin and Salzburg investigated the effectiveness of *Iyengar Yoga* in patients with chronic neck pain. The study was first published with low patient enrolment (*n* = 55 patients) [27], then updated results were published [57]. In the latter study, patients (*n* = 77) were enrolled and randomly assigned to a 90-min *Iyengar Yoga* class once a week over 9 weeks (*n* = 38) or to a self-care exercise program. Yoga led to higher pain relief, with possible additional effects on psychological well-being and quality of life [57].

In another clinical study, patients with chronic neck pain (*n* = 56) were randomly assigned to three groups, namely pilates (*n* = 20), *āsana Yoga* (*n* = 18), and isometric exercises (*n* = 18). *Iyengar Yoga* included two sets of 10 repetitions per day of four *āsana* (10–20 s each). All groups performed 6 weeks of exercises. Pain, disability, depression, and quality of life improved with a similar trend in all groups [58].

Guidelines from several American universities (USA, Canada, Chile) recommend treating neck and lower back pain with non-invasive interventions. The study emphasizes self-care, education and non-pharmacological therapies, particularly those that “actively” focus on movement and on addressing psychological/social factors associated with pain [59].

In our proposed Yoga protocol for neck pain we proposed and showed movements to reduce tightness in the different muscular bone ties responsible for neck pain and cervical disc compression in individuals with forward head posture (protruded head). In detail, the stretches for semispinalis capitis, levator scapula, and suboccipital muscles (neck flexion shown in Figure 2a,f–j, Figure 3e and Figure 6e), and for sternocleidomastoid, scalene, and hyoid muscles (neck extension illustrated in Figure 2a–e, Figure 3a–c,f–j and Figure 6a; neck twists displayed in Figure 4c–e,g,i, Figure 5c,h, Figure 7a–h and Figure 8a–h) were described.

We would highlight that our university Yoga program at the dental school include a large number of *āsana* and movements for the neck in supine (*Uttāna*), prone (*Nyubja*), and standing (*Utthāna*) positions, also using props, supports, and ropes (*Rajju) āsana* and following *Hatha Yoga* technique and its evolutions (*Iyengar* and *Parināma*).

In our proposed Yoga protocol we conceived exercises to prevent the loss of physiological cervical lordosis or its recovery and *āsana* for the release of tight and retracted anterior neck muscles for the prevention of outlet syndromes.

We also use specific *Yoga Therapy* protocols to treat occlusion issues in the presence of spine-related postural alterations.

### 4.9. Upper Syndromes 

#### 4.9.1. Upper Crossed Syndrome in Dentists

We also need to illustrate that the working position of dental professionals favors the development of upper crossed syndrome (visually describable as rounded shoulders and a depressed/indented chest), which refers to a deleterious posture caused by muscle imbalances in the neck and upper back regions (weakening vs. lengthening, anterior vs. posterior muscles, upper back vs. neck muscles) [60]. Specifically, tight suboccipitals, upper trapezius, and levator scapulae on the dorsal side associated with weak deep cervical flexors, crossed with tight pectoralis and sternocleidomastoid on the anterior side associated with weak rhomboids, and lower trapezius and serratus anterior, create an impaired posture. This imbalanced pattern causes joint dysfunctions, particularly at the atlanto-occipital joint, C4–C5 segment, cervicothoracic joint, glenohumeral joint, and T4–T5 segment. 

#### 4.9.2. Thoracic Syndromes among Dentists

Thoracic outlet syndromes result from compression of the brachial plexus, including the brachial nerves (neurogenic syndrome), subclavian artery (arterial syndrome), and/or subclavian vein (venous syndrome) in the thoracic outlet region [61].

The anatomical path of the nerve bundle (the lower four cervical nerves and the first thoracic nerve emerging from the spinal cord) and subclavian artery (from the dorsal aorta, then forming brachial arteries) passes through the scalene triangle space (between the anterior and median scalene muscles in the neck), through the thoracic outlet space (between the clavicle/collarbone and first rib), under the pectoralis minor space, then into the armpit and down the arm. Brachial veins (from the brachiocephalic vein and then subclavian vein) pass through the thoracic outlet space and pectoralis minor space.

The authors need to deliver the concept that tight scalene muscles for chronic neck tension, retracted posterior neck muscles, head protrusion (forward head posture), rounded back, depressed chest, sloped shoulders, and weak shoulder muscles, i.e., all the muscle-related problems caused by poor posture as in the case of dental professionals, can cause outlet compression and trigger outlet syndromes.

Brachial nerves and blood vessels provide motor control, sensitivity, and blood to the chest, shoulder, arm, and hand. Symptoms of outlet syndromes are pain (in the neck, shoulder, or arm); numbness or tingling, weakness or fatigue, lack of color and cold fingers in the arm or hand; weak or absent pulse, and swelling in the arm. Repetitive movements over a long period cause muscle stiffening with minor trauma, inflammatory processes and swelling and therefore triggering the compression of the musculoskeletal structures. Compression points are interscalene triangle (anterior to middle *scalene muscles*), costoclavicular space (clavicle and *subclavius muscle* to the first rib) and subpectoral space (third–fifth ribs to the *pectoralis minor muscle*) [62].

There is no literature specifically referring to thoracic outlet syndromes among dental professionals (likely as they are included among shoulder pathologies), and no literature on the use of yoga to treat or prevent symptoms or the onset of pathology.

In our proposed Yoga protocol for the shoulders we describe *āsana* and their action to elucidate their effect on the prevention and treatment of compressive thoracic disorders such as thoracic outlet syndrome, hyperkyphosis, and chest depression.

We focused on the mobilization of the thoracic spine, dorsal kyphosis, sternoclavicular joint, and involved muscles such as the *subclavius*, *deltoids*, *pectoralis*, *trapezius* and *sternocleidomastoids*.

We highlighted that mobilization and extension of the thoracic spine are fundamental for physiological diaphragm movement during breathing and the related functionality of the abdominal organs otherwise compressed by hyperkyphosis, rounded shoulders and back, and depressed chest.

### 4.10. Shoulder Pain in Dentists

A previous review from the University of Connecticut reported that shoulder problems affect 20–65% of dentists, 35–76% of dental hygienists and 27–62% of dental assistants [11].

A survey-based study considering dental operators (*n* = 45) reported a high prevalence of shoulder pain and disorders among dental practitioners [63]. Most of the dentists (75.9%) and dental assistants (90.9%), as well as nearly half of the dental technicians (40%) experienced discomfort in areas of the neck and shoulders.

Another survey-based study on Australian dentists (*n* = 283) reported that 55% of them presented with MSDs of the shoulder, and, in 21.8% of these, the MSDs interfered with their daily routine [64].

A survey-based study from the University of Iowa on dental hygienists (*n* = 95) reported that 60% of them suffered from job-related MSDs of the shoulder [65].

#### Yoga vs. Shoulder Disorders

A randomized study from a Turkish university examined the effects of yoga on shoulder and arm pain and physical performance in patients with breast cancer. The patients (*n* = 42) were randomly assigned to a 10-week *Hatha Yoga* (1 h lesson/2 days a week for 10 weeks) course (*n* = 21) or to 10-week activity including a booklet for at home exercises (stretching and breathing) (control *n* = 21). The patients in the yoga group experienced an improvement in shoulder and arm pain severity when compared to the control group [66].

A recent prospective randomized controlled trial from an Indian University on patients with frozen shoulders analyzed the effect of *āsana* (from Swami Satyananda Saraswati book, 2000) in addition to conventional therapy. Patients received a 4-week yoga-at-home course (30 min per day every week) (*n* = 36) or no intervention (*n* = 36), even though both groups received NSAIDS and physical therapy. In both groups, a significant reduction in shoulder pain and shoulder disability scores was observed [67].

In our proposed Yoga protocol for the shoulder we display and elucidate in depth the execution of different *āsana*, and described their beneficial effect for the prevention and treatment of compressive disorders such as shoulder impingement, thoracic outlet syndrome, hyperkyphosis, and chest depression.

## 5. Conclusions

Yoga, as an integrative science in medicine and public health, represents a powerful tool for the prevention and treatment of occupational musculoskeletal disorders and an extraordinary path for the self-care of dental professionals, sitting job workers, and healthcare providers suffering from occupational biomechanical stresses and awkward postures.

This paper represents a guideline for self-cure in the prevention or treatment of musculoskeletal disorders affecting dental professionals.

Yoga is a powerful concentrative self-discipline able to provide physical and mental well-being, representing great help and support in daily life and business for dental professionals. *Yógāsana* restores retracted and stiff muscles, giving relief to the strained and tired limbs of dental professionals. Yoga is not intended for flexible or physically performing persons but for people who decide to take care of themselves.

The practice of specific *āsana* represents a powerful tool for the prevention or treatment of MSDs related to poor posture, forward head, chronic neck tension (and related headache), depressed chest, compressive disorders on wrists and shoulders as carpal tunnel, impingement syndromes, outlet syndrome, subacromial pain syndrome and spinal disc pathologies. 

We are available to bring our expertise and knowledge in academic contexts and to spread and share our targeted Yoga protocols with students, faculties and academics in the universities around the world. 

## Figures and Tables

**Table 1:** Working posture for dental professionals *.

✓ **Sitting posture** on pelvis ischial tuberosity, with a slight anterior pelvic tilt ** (sacral nutation) and maintaining the lumbar lordosis and equally bearing weight on the pelvis, ** maintain light core activation (deep abdominals mainly transversus abdominis and pelvic floor)
✓ Straight **back** avoiding rounding into a “C” shape, ** maintain the anti-gravity thrust of the spine, ** keep diaphragmatic breathing
✓ **Trunk** forward inclination ≤ 20°, trunk-leg angle ≥ 90°
✓ **Head** forward inclination ≤ 20–25°; head tilt (twisted or other positions) ≤ 15°
✓ ** Relaxed **shoulders**, depressed and adducted **scapulae**
✓ **Arms** along the body, forward-oriented ≤ 10°, abduction ≤ 25°
✓ **Forearms** raised in flexion ≥ 90° from the horizontal line
✓ Thigh-calf angle 90–110°
✓ Angle between thighs ≤ 45°
✓ **Calves** perpendicular to the floor
✓ **Feet** on the floor and forward-oriented
✓ Regular rest **breaks** and stretching/therapeutic exercises between working hours
✓ Training to improve posture (to reduce forward head posture and crossed syndromes)

* elaboration targeted to postural dental ergonomics based on the rationale of yòga-therapy and ESDE 2007; ISO 11226:2000/Cor 1:2006; EN 1005-4, ISO 6385:2004; Postural Guidelines of the Italian Ministry of Health 2018 recommendations; ** Yoga-based postural guidelines (Gandolfi unpublished data).

## Data Availability

Data sharing not applicable.
